# Effect of JAK Inhibitors on Pain Management in Patients with Rheumatoid Arthritis: A Literature Review

**DOI:** 10.3390/jcm15145348

**Published:** 2026-07-08

**Authors:** Aleksandra Kowalska, Aleksandra Jawoszek, Aleksandra Borkowska, Grzegorz Chmielewski, Łukasz Jaśkiewicz, Magdalena Krajewska-Włodarczyk

**Affiliations:** 1 Department of Mental and Psychosomatic Diseases, School of Medicine, Collegium Medicum, University of Warmia and Mazury in Olsztyn, 10-719 Olsztyn, Poland; olajawo@gmail.com (A.J.); al.borkowska14@gmail.com (A.B.); gchmielewski.gc@gmail.com (G.C.); 2 Department of Human Physiology and Pathophysiology, School of Medicine, Collegium Medicum, University of Warmia and Mazury in Olsztyn, 10-082 Olsztyn, Poland; lukasz.jaskiewicz@uwm.edu.pl

**Keywords:** RA, rheumatoid arthritis, tsDMARD, JAKi, JAK inhibitors, pain

## Abstract

The introduction of biologic disease-modifying antirheumatic drugs (bDMARDs) and targeted synthetic disease-modifying antirheumatic drugs (tsDMARDs) has significantly enhanced the prognosis for patients with rheumatoid arthritis (RA). Nevertheless, improving quality of life remains a major clinical challenge requiring the implementation of multidirectional therapeutic measures. Pain reduction plays a particularly important role in this context, representing one of the primary treatment goals for many patients. Scientific evidence indicates that effective pain management is a crucial predictive factor for the improvement of mental and physical health in patients with RA. The aim of this literature review was to summarise the mechanisms of action of Janus kinase inhibitors (JAKi), with particular emphasis on their modulation of pain generation and transmission. Furthermore, the study aimed to compare the results of clinical studies and assess the actual effectiveness of these drugs in reducing pain in clinical practice. JAKi exhibit peripheral analgesic effects by directly reducing the production of pro-nociceptive cytokines and modulating macrophage polarisation. Moreover, they influence central pain processing mechanisms by modulating the IL-6/JAK/STAT3 pathway and by reducing microglial and astrocyte proliferation in the dorsal horn of the spinal cord. The results of randomised clinical trials confirm that JAKi provide rapid, clinically significant pain reduction. Some studies also point to the persistence of this effect over a longer period, and to their greater efficacy compared with conventional disease-modifying antirheumatic drugs (cDMARDs) and bDMARDs. The efficacy of this group of drugs has also been noted in patients with an inadequate response to prior therapy with biological drugs. A key focus of future research remains determining the optimal timing for introducing JAKi in the treatment of RA and identifying predictive factors to enable the selection of patients most likely to benefit from this class of drugs.

## 1. Introduction

Rheumatoid arthritis is a chronic, systemic inflammatory autoimmune disease of unknown etiology [1,2]. The disease process most commonly leads to symmetrical involvement of peripheral joints, manifesting as joint pain and swelling accompanied by morning stiffness, resulting in reduced patients’ functional capacity and diminished quality of life [1,2,3,4]. The disease may also manifest with extra-articular symptoms, such as rheumatoid nodules, pericarditis, and pulmonary fibrosis [5].

The disease significantly reduces quality of life by adversely affecting not only physical health but also mental well-being and patients’ daily functioning.

Over the years, a number of questionnaires and scales have been developed to enable a holistic approach to the problems faced by patients with RA. These include, inter alia, Short Form 36 Health Survey (SF-36), which assesses quality of life, The Health Assessment Questionnaire—Disability Index (HAQ-DI), which assesses physical functioning and capacity, and the Patient Global Assessment of Disease Activity (PtGA), which assesses disease activity from the patient’s perspective. In clinical practice, these scales objectively identify the factors that most significantly impact patients’ quality of life. At the same time, they serve as a reliable indicator of the efficacy of the introduced treatment, enabling an assessment of its actual impact, including its effects on key areas that determine quality of life. Considering the patient’s quality of life as a measure of RA treatment efficacy is important, as it is directly correlated with both adherence to therapeutic recommendations and satisfaction with treatment [6].

One of the important elements of RA treatment, which significantly affects patients’ quality of life and determines the efficacy of treatment, is the relief of pain [6,7]. The importance of pain reduction in this group of patients was confirmed by a multicentre study conducted by Vibeke Strand and Grace C., which demonstrated that 70% of participants reported the primary goal of treatment was to reduce pain severity. At the same time, as many as 63% of the participants experienced pain on a daily basis, and 75% regularly used analgesics [7]. This is also confirmed by the fact that pain is recognised as the most significant predictor of both mental and physical health in patients with RA [8].

The introduction of biological drugs and biosimilar disease-modifying antirheumatic drugs (bDMARDs) as well as targeted synthetic drugs (tsDMARDs) has enabled a treatment focused on the immune pathways involved in the pathophysiology of the disease [6]. Not only do these drugs reduce inflammation and prevent structural joint damage, but they also enable effective control of the disease, leading to either low disease activity or clinical remission [6,9]. The tsDMARDs group includes drugs in the JAK/STAT pathway inhibitor class. The European Medicines Agency (EMA) and the US Food and Drug Administration (FDA) have approved several JAKi for the treatment of RA. These include tofacitinib (JAK1/JAK3 inhibitor), baricitinib (JAK1/JAK2 inhibitor), upadacitinib (JAK1 inhibitor), and filgotinib (JAK1 inhibitor). In Japan, peficitinib (pan-JAK inhibitor) has also been approved for the treatment of RA [10].

In recent years, numerous scientific reports have described the mechanisms of action of JAKi and their role in modulating signalling pathways crucial to the pathogenesis of pain in RA. At the same time, numerous clinical studies were published, which assessed the clinical efficacy of this class of drugs in reducing pain in patients with RA. This literature review summarises the current state of knowledge on the pharmacodynamics of JAKi and their impact on pain management in clinical practice.

## 2. Mechanism of Action of JAKi

JAKi are a breakthrough class of orally administered targeted synthetic disease-modifying antirheumatic drugs. They exhibit a broad spectrum of anti-inflammatory action, with the ability to simultaneously block the intracellular signalling of multiple cytokines. Unlike bDMARDs, which act selectively on individual cytokines, JAKi enable a more comprehensive modulation of the inflammatory response [11].

The Janus kinase/Signal Transducers and Activators of Transcription (JAK/STAT) signalling pathway is one of the most important mechanisms of intracellular communication. It is involved in the transmission of signals from over fifty cytokines and growth factors, such as hormones, interferons, interleukins, and cell colony-stimulating factors. Janus kinases remain non-covalently bound to cytokine receptors [12]. Each of these receptors comprises an extracellular cytokine-binding domain and an intracellular cytoplasmic region that interacts with one or more JAK-family kinases. This family consists of four isoforms: JAK1, JAK2, JAK3 and TYK2 (tyrosine kinase 2) [13]. The specific nature of signal transmission in the JAK/STAT pathway is determined by the type of cytokine receptor complex and its affinity for individual JAK kinases [14]. After the ligand binds to the receptor, kinases are activated, which phosphorylate receptor tyrosine residues, thereby enabling the binding of STAT proteins [12]. Activation of the receptors leads to the phosphorylation of proteins in the STAT family, which act as transcription factors. In mammals, seven members of this family have been described: STAT1, STAT2, STAT3, STAT4, STAT5a, STAT5b and STAT6. Each of these can be activated by various type I and II cytokine receptors and their associated JAK kinases, providing a high degree of complexity and precision in regulating the cellular response [13]. The activation of the JAK/STAT pathway affects a number of physiological functions, including haemopoiesis, the immune response, tissue regeneration, control of the inflammatory response, apoptosis and adipogenesis [12].

In RA, activation of the JAK/STAT pathway is among the key mechanisms that sustain and intensify the chronic inflammatory condition [15]. In this process, interleukin 6 (IL-6) plays a key role: by activating the JAK/STAT signalling pathway, it stimulates the production of IL-1 and TNF-α and promotes the differentiation of T lymphocytes into the Th17 subpopulation, which is responsible for the synthesis of IL-17. In the course of RA, elevated levels of IL-6 are observed. By binding to the gp130 receptor, IL-6 induces the phosphorylation of STAT1 and STAT3 proteins, mediated by JAK1 and JAK2 kinases. This process is essential for cell differentiation, proliferation, and osteoclastogenesis. In the pathogenesis of rheumatoid arthritis, a special role is attributed to STAT3, whose activity promotes angiogenesis, increases fibroblast survival by inhibiting apoptosis, and increases the expression of selected matrix metalloproteinases, including MMP-2 and MMP-9, which are responsible for the degradation of joint structures [16]. Another significant cytokine in the pathogenesis of RA is IL-23, which is produced by dendritic cells and activated macrophages. It binds to its receptor (IL-23R/IL-12Rβ1), activating the JAK2/TYK2 pathway, followed by the STAT3/STAT5 pathway. IL-23 promotes the differentiation and maintenance of Th17 lymphocytes, which secrete IL-17, thus enhancing the production of pro-inflammatory cytokines, namely TNF-α, IL-1β, GM-CSF, PGE2 and chemokines. Furthermore, IL-23 increases MMP concentration in synovial fluid, resulting in cartilage destruction, osteoclastogenesis, and the progression of inflammation [17]. A high concentration of IL-23 correlates with disease activity and is therefore considered a key component of the IL-23/Th17/IL-17 axis and a potential biomarker for RA [18]. The link between TNF-α and the JAK-STAT pathway in synovial fibroblasts is based on the mechanism of indirect, delayed activation. TNF-α not only directly activates the JAK-STAT signalling pathway but also induces the expression of secondary mediators, in particular IFN-β and IL-6. The key element of this mechanism is the activation of the interferon response factor 1 (IRF1), which, upon TNF-α stimulation, increases transcription of the IFNB1 gene. The secreted IFN-β then acts in the autocrine mechanism through the type I interferon receptor (IFNAR), leading to the activation of JAK1 and JAK2 kinases, and the delayed phosphorylation of the STAT1 protein, which occurs 3–4 h after the TNF-α stimulation. The consequence of this cascade is the induction of a characteristic signature of interferon response genes, including, e.g., the expression of chemokines such as the BAFF factor, CXCL9 and CXCL10 [19].

JAKi exert their action by intracellularly binding to the kinase domains (JH1) in a manner that competes with adenosine triphosphate (ATP), thus preventing the transfer of phosphate groups onto signalling proteins. Consequently, the activation of STAT proteins is inhibited, and the cytokine signal is disrupted, leading to reduced expression of pro-inflammatory genes. A key pharmacological feature of JAKi is their varying selectivity for individual isoforms, which is determined by dose and tissue type [20]. JAKi include pan-inhibitors (baricitinib, tofacitinib, peficitinib), which act on multiple isoforms, and selective inhibitors of the JAK1 isoform (upadacitinib, filgotinib) [21]. The results of in vitro studies have enabled a quantitative comparison of the activity of individual JAKi. Biochemical tests using isolated enzymes showed that pan-inhibitors effectively inhibited JAK1, JAK2 and JAK3 isoforms, with baricitinib exerting a weaker effect on JAK3. In turn, upadacitinib and filgotinib, in addition to their activity against JAK1, exhibited significant inhibitory activity against JAK2 [22,23]. To determine how differences in the strength of action of individual JAKi observed in vitro translate into their activity against specific cytokine pathways in vivo, an integrated analytical model was used. The study combined data from in vitro tests conducted in whole blood with plasma pharmacokinetic analysis, enabling the determination of JAK-dependent cytokine receptor inhibition profiles for tofacitinib, baricitinib, upadacitinib and filgotinib at concentrations corresponding to clinically effective doses used in patients with RA. The obtained results indicate that, despite observed differences in in vitro activity, cytokine receptor inhibition profiles were comparable among the individual inhibitors after accounting for plasma protein binding, the blood-to-plasma concentration ratio, and clinical exposure. However, minor quantitative differences were also noted. Tofacitinib inhibited cytokine receptors activated by the JAK1/JAK3 complex (IL-2, IL-4, IL-7 and IL-15) more potently, whereas baricitinib exhibited a higher degree of inhibition of receptors associated with the JAK2/TYK2 pair (IL-12, IL-23 and erythropoietin). In contrast, upadacitinib exhibited greater activity against JAK1/JAK2-dependent receptors (IFN-γ, G-CSF) and JAK2-dependent receptors (thrombopoietin, IL-3, GM-CSF) [24].

## 3. Effect of JAKi on Pain Management in Patients with RA

According to the definition proposed by the International Association for the Study of Pain (IASP), pain is defined as an unpleasant sensory and emotional experience associated with actual or potential tissue damage, or described in terms of such damage [25].

### 3.1. The Pathogenesis of Pain in RA, and the Potential Role of JAKi in Its Modulation

The pathomechanism of pain in RA comprises three components: local inflammation, peripheral sensory mechanisms, and abnormal pain regulation in the central nervous system [26].

#### 3.1.1. Local Inflammation

In the synovial membrane affected by an inflammatory process, pain mediators (algogens) are released, such as kinins, pro-inflammatory cytokines: interleukin 1 (IL-1), IL-6, tumour necrosis factor alpha (TNF-alpha), neuropeptides, such as calcitonin gene-related peptide (CGRP), and neurotrophins (nerve growth factor, NGF) [27]. Monocytes found in the affected joint differentiate into the pro-inflammatory M1 phenotype [28,29]. The NGF factor promotes the transformation of macrophages (M0) into M1 macrophages. It also stimulates their degranulation, leading to the release of histamine, which in turn stimulates nociceptors [27,29]. Within the nociceptor endings, there are voltage-gated and ligand-gated ion channels—transient receptor potential vanilloid type 1 (TRPV1), transient receptor potential ankyrin 1 (TRPA1), and sodium voltage-gated channel alpha subunit 9 (Nav1.7) [30]. Pro-inflammatory cytokines activate these channels, leading to the sensitisation of nociceptive neurons [27,30]. In turn, activated nociceptors release neuropeptides that modulate the functions of innate and adaptive immune cells [30].

These phenomena result in an increase in the number of nociceptive fibres under the influence of growth factors found in the synovial membrane. Cytokines, by acting on ion channels, alter the properties of the neuronal cell membrane, lowering the action potential threshold and increasing sensitivity to stimuli [30].

#### 3.1.2. The Central Mechanism of Pain

Dysregulation of pain transmission pathways within the CNS and central sensitisation at the spinal cord level are responsible for the experience of chronic pain in patients with RA [27,31,32]. During the course of the disease, descending pathways that inhibit pain transmission are reduced, while those that facilitate pain transmission are increased. Recent studies suggest that disturbances within the CNS influence patients’ pain perception to a similar extent as a local inflammatory condition [31,32]. A study using the Central Sensitisation Inventory (CSI) questionnaire, which assesses the role of central sensitisation in pain perception in patients with RA, demonstrated that this mechanism accounts for 41% of the intensity of the experienced pain [33]. Hence, even though patients with RA have a well-controlled inflammatory condition, they may still experience pain [31].

#### 3.1.3. The Proposed Mechanism of Action of JAKi in Reducing the Perceived Pain

JAKi bind to JAK kinases and inhibit the activation of the JAK/STAT pathway. Drugs in this group can reduce perceived pain both by directly inhibiting the production of pro-nociceptive cytokines signalled via this pathway, and by regulating macrophage polarisation [34,35,36]. The inhibition of the JAK/STAT pathway reduces the production of pro-inflammatory cytokines, such as IL-6, IL-7, IL-15, IL-21, IFN-α and IFN-β, leading to the suppression of the inflammatory response [35]. Moreover, the use of JAK2/STAT3 inhibitors may facilitate the transition of macrophages from the M1 (pro-inflammatory) phenotype to the M2 (anti-inflammatory) phenotype. This process is associated with a decrease in the expression of pro-inflammatory mediators (IFN, TNF-α, IL-1β, IL-6, nitric oxide synthase (iNOS)), and an increase in the production of anti-inflammatory factors (IL-10, transforming growth factor (TGF-β), insulin-like growth factor (IGF-1), vascular endothelial growth factor (VEGF), epidermal growth factor (EGF), and platelet-derived growth factor (PDGF)) [36,37]. A reduction in pro-inflammatory cytokine concentration affects the activity of nociceptive neurons, both directly and indirectly. This mechanism involves a decrease in the activation of afferent fibres, resulting from reduced interaction of pro-inflammatory cytokines with receptors on sensory neurons. Additionally, secondary signalling pathways are modulated, which reduces the excitability of cells transmitting pain stimuli to the dorsal horn of the spinal cord, and, as a result, reduces the perception of pain [35].

In the context of chronic pain, studies using animal models have demonstrated that peripheral nerve injury activates the JAK/STAT3 pathway in dorsal root ganglion (DRG) microglia [35,38]. This process leads to increased IL-6 concentration in the spinal cord [35]. This cytokine is crucial in the modulation of nociception, as spinal cord cells, namely neurons, glial cells, and dorsal root ganglia (DRG), express the IL-6 receptor (IL-6 R) and the gp130 protein, making them sensitive to IL-6 signalling [15,35].

Cytokines produced in the DRG can be transported to the central nervous system through afferent neuron terminals, where they activate the JAK2/STAT3 microglial pathway, inducing neuropathic pain. Activated microglia and neurons release pro-inflammatory mediators, such as prostaglandins, reactive oxygen species, pro-inflammatory factors, nitric oxide, and ATP, which additionally intensify the transmission of pain stimuli [39].

Furthermore, activation of the JAK/STAT3 pathway is also responsible for astrocyte proliferation, leading to an intensified neuroinflammatory response and increased production of pro-inflammatory mediators in the dorsal roots of the spinal cord [35]. Studies using animal models have demonstrated that inactivation of the JAK/STAT3 pathway in spinal cord glial cells by expression of the cytokine signalling suppressor SOCS3 (a physiological protein inhibitor of JAK/STAT3) prevented abnormal IL-6 expression and significantly reduced the severity of mechanical allodynia in rats [35,38].

It was also demonstrated that baricitinib (JAK1/2 inhibitor) reduced inflammatory and neuropathic pain by modulating the IL-6/JAK/STAT3 pathway, decreasing colony-stimulating factor 1 (CSF-1) expression in DRG neurons, and limiting microglial and astrocyte proliferation in the dorsal horn of the spinal cord [15].

**Figure 1:**
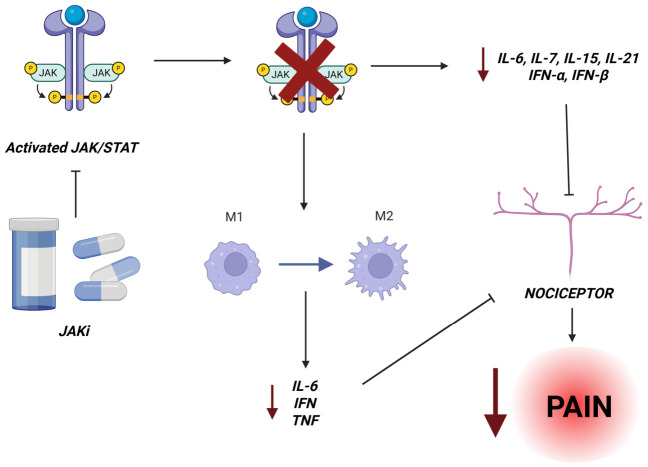
The proposed mechanism of the analgesic action of JAKi. Red ↓ decrease. Created in BioRender. Jaskiewicz, L. (2026) https://BioRender.com/5tv64y5.

Figure 1 illustrates the above-described mechanism of the analgesic action of JAKi.

JAKi inhibit the activation of the JAK/STAT signalling pathway, leading to decreased production of pro-inflammatory cytokines, such as IL-6, IL-7, IL-15, IL-21, IFN-α, and IFN-β. Inhibition of this pathway also promotes the transition of macrophages from the M1 to the M2 phenotype, leading to decreased production of pro-inflammatory mediators, including IL-6, IFN and TNF. As a result, both mechanisms contribute to reduced nociceptor activation and decreased pain transmission.

List of abbreviations: JAKi—Janus kinase inhibitors; IL-6—interleukin 6; IL-7—interleukin 7; IL-15—interleukin 15; IL-21—interleukin 21; IFN-α—interferon alpha; IFN-β—interferon beta; and TNF—tumour necrosis factor.

### 3.2. An Assessment of the Effect of Selected JAKi on Pain Management in Patients with RA

#### 3.2.1. Baricitinib

A study comparing the efficacy of baricitinib monotherapy or baricitinib in combination with methotrexate (MTX) against MTX monotherapy observed a statistically significant improvement in pain control when using the VAS. A higher percentage of patients receiving baricitinib as monotherapy (40.9%), or baricitinib in combination with MTX (42.1%), compared with patients receiving MTX (23%), achieved a pain threshold equivalent to the absence of pain (VAS < 10 mm) by the end of the study [40].

A post hoc analysis of three phase 3 randomised studies (RA-BEAM, RA-BUILD, RA-BACON) showed that the introduction of baricitinib at a dose of 4 mg daily into treatment, in both opioid-using and non-opioid-using patient groups, improved pain control (assessed using the VAS) to a greater extent as early as 1 week after initiation of treatment with the drug, and at subsequent time points, compared with the placebo group. At the end point of this study (week 24), it was shown that, in opioid users, the use of baricitinib at the above dose reduced pain by 13.4 points, as measured on the VAS, compared with placebo. In contrast, in non-opioid users, the introduction of baricitinib reduced pain by 14.3 points, as measured on the VAS, compared with placebo. The use of baricitinib at a lower dose (2 mg daily) also reduced pain more effectively than a placebo, with this effect observed by week 4 of treatment [41].

#### 3.2.2. Filgotinib

A study comparing the effect of filgotinib on pain management versus a placebo, adalimumab or methotrexate, found a significantly greater reduction in pain, particularly in patients receiving a daily dose of 200 mg of this drug. The introduction of this drug shortened the time to first reported pain reduction compared with other drugs used in this study, with pain reduction assessed using the VAS noted as early as 2 weeks after drug initiation. When comparing the efficacy of filgotinib with that of adalimumab, more effective pain management was observed at weeks 2 and 4 of treatment with a 200 mg dose, whereas filgotinib at 100 mg was comparable to adalimumab [42,43].

#### 3.2.3. Tofacitinib

A post hoc analysis involving four phase 3 randomised clinical studies, ORAL Scan, ORAL Sync, ORAL Standard, and ORAL Step, showed that the use of tofacitinib at a dose of 5 mg or 10 mg twice daily significantly reduced pain severity as assessed on the PAAP scale (improvement threshold ≥ 20 mm on the PAAP-VAS scale), compared with a placebo. The results obtained using the SF-36v2 questionnaire confirmed both a significant and rapid improvement in pain management: in patients following unsuccessful treatment with TNF-α inhibitors, the effect was observed as early as week 2, whereas in patients following unsuccessful treatment with cDMARDs, it was observed within month 1. Importantly, the achieved improvement was maintained up to month 6 of a follow-up study [44].

A post hoc analysis of nine randomised studies (six phase 3 clinical studies and one III/IVb phase study: ORAL Step, ORAL Scan, ORAL Solo, ORAL Sync, ORAL Standard, ORAL Start and ORAL Strategy), assessing the management of joint pain after 3 months of treatment, found that the percentage of patients in whom an at least 50% reduction in VAS scores was observed, was 67.4% for tofacitinib, 65.1% for adalimumab, and 40.0% for placebo.

The analysis demonstrated that some patients in the absence of active inflammation continued to experience residual pain. In these patients, the introduction of tofacitinib or adalimumab also reduced perceived pain; the percentage of patients achieving a VAS score < 20 was 55.7% with tofacitinib, and 51.7% with adalimumab, compared with 40% in the placebo group [45].

#### 3.2.4. Upadacitinib

A post hoc analysis of the SELECT-NEXT study showed that in patients with RA with an inadequate response to at least one of the following cDMARDs: MTX, leflunomide or sulphasalazine, the introduction of upadacitinib at doses of 15 mg and 30 mg, compared with placebo, was associated with a significant reduction in pain as early as week 1 of treatment, whereas in the placebo group, a comparable response was noted in week 4 of treatment [46]. Furthermore, a post hoc analysis using data from the phase III SELECT-BEYOND study showed that in patients with RA with an inadequate response to bDMARDs, the median time to achieve a significant reduction in pain was 2 weeks in the group treated with upadacitinib (15 mg or 30 mg) while continuing treatment with cDMARDs, compared with 4 weeks in the placebo group [47]. Upadacitinib at doses of 15 mg and 30 mg was associated with a statistically significant reduction in pain (NNTs = 3–4) in week 12 after the introduction of the drug [46,47]. In addition, the SELECT-BEYOND study, which used the SF-36 questionnaire, demonstrated that 12 weeks after the introduction of upadacitinib (15 mg or 30 mg), 24% of patients achieved scores in the pain domain comparable to those of the general population, whereas this proportion was 11% in the placebo group [47].

The SELECT COMPARE study, which compared upadacitinib (15 mg once daily) with adalimumab (40 mg every two weeks), demonstrated that after 12 weeks of treatment, the proportion of patients receiving upadacitinib, who achieved scores in the pain domain of the SF-36 questionnaire comparable to those of the general population, was significantly higher than in the group receiving adalimumab (*p* < 0.05). Furthermore, using the VAS, it was demonstrated that in weeks 26 and 48 of treatment, patients receiving upadacitinib experienced a significantly greater reduction in pain than those receiving adalimumab (*p* < 0.05) [48].

Table 1 summarises the characteristics and the results of the studies considered in the present review.

### 3.3. Clinical Applications of JAKi

In routine clinical practice, and in accordance with current EULAR recommendations, JAKi are not positioned as first-line therapy in RA. Rather, they are considered an advanced therapeutic option in patients who do not achieve adequate disease control with csDMARDs. In this setting, treatment escalation with either a bDMARD or tsDMAR, including JAKi, is recommended [49].

The 2021 ACR recommendations are consistent with this therapeutic approach. In these guidelines, JAKi are classified as tsDMARDs, and this group includes tofacitinib, baricitib, and upadacitinib. In DMARD-native patients with moderate-to-high disease activity, the ACR strongly recommends methotrexate over bDMARD or tsDMARD monotherapy. This preference reflects the established role of MTX as a first-line agent, its well-characterized and safety profile, lower treatment cost, and existing safety concerns regarding JAK inhibitor therapy [50].

Importantly, JAKi, similarly to agents targeting the interleukin-6 pathway, may be particularly useful in patients who are unable to receive concomitant csDMARD therapy, given their demonstrated efficacy as monotherapy. Nevertheless, the decision to initiate treatment with a JAKi should be based on an individualized benefit–risk assessment, with particular attention to cardiovascular, malignancy-related, and thromboembolic risk factors [49].

The introduction of JAKi into subsequent lines of therapy has expanded the therapeutic options available for patients with an inadequate response to previous disease-modifying treatment [51]. Evidence from real-world registries supports their effectiveness in routine clinical practice and indicates that they may represent a clinically relevant therapeutic alternative in patients requiring escalation during the course of disease management [52].

Persistent pain despite ongoing treatment remains a significant clinical challenge in patients with RA and may be associated with the coexistence of fibromyalgia or mechanisms of central sensitization [53]. The prevalence of fibromyalgia among patients with RA has been estimated to range from approximately 5% to 52% while features of central sensitization may be present in up to 40% of patients [15,54]. These conditions may substantially amplify pain perception and affect the assessment of disease activity, potentially leading to an overestimation of composite disease activity indices that incorporate patient-reported outcomes. Therefore, before intensifying anti-inflammatory therapy, it is important to distinguish between persistent inflammatory activity and pain driven by non-inflammatory mechanisms [50,53]. Although JAKi have been shown to exert beneficial effects on pain perception, including pain components related to central sensitization, the available evidence does not support the coexistence of fibromyalgia or altered pain-processing mechanisms as an independent rationale for the earlier or preferential use of this class of agents [49,55].

The therapeutic utility of JAKi extends beyond RA, encompassing a broad spectrum of immune-mediated inflammatory diseases. The expanding range of indications for this class of agents reflects the shared pathogenic mechanisms underlying many autoimmune and immune-mediated disorders. Despite their diverse clinical manifestations, these conditions are frequently driven by common inflammatory pathways involving cytokines that signal through the JAK-STAT cascade. Given the central role of JAK-STAT signaling in the transduction of multiple cytokine-mediated inflammatory responses, pharmacological inhibition of this pathway offers a rational therapeutic strategy for modulating aberrant immune activation across various disease states. Consequently, JAKi have demonstrated efficacy not only in RA but also in several other conditions characterized by chronic immune-mediated inflammation [56].

The most extensive clinical evidence has been generated for tofacitinib, whose efficacy has been established in psoriasis, psoriatic arthritis, and ulcerative colitis. Favorable therapeutic outcomes have also been reported for baricitinib in patients with moderate-to-severe psoriasis, atypical neutrophilic dermatosis with lipodystrophy, and systemic lupus erythematosus. In addition, an increasing amount of evidence suggests the potential therapeutic utility of JAKi in disorders characterized by dysregulated type I interferon signaling, including systemic lupus erythematosus and polymyositis [56].

Collectively, these findings suggest that agents within this therapeutic class may contribute to the development of more effective targeted treatment strategies for autoimmune diseases.

## 4. Summary

Pain perception in patients with RA is a key factor affecting both mental health and physical functioning. Therefore, effective pain management should be an integral part of the therapeutic approach.

The available data suggest that JAKi may represent an effective treatment option for patients with severe pain. This group of drugs is particularly important for patients who have not achieved a satisfactory level of pain management despite treatment with csDMARDs and bDMARDs. The mechanism of action of JAKi suggests they may modulate pain by directly influencing inflammatory processes in peripheral tissues and by modulating central mechanisms of pain stimulus processing. However, it should be emphasized that current knowledge regarding the effects of drugs from this group on the mechanisms underlying pain development in humans is limited. Much of the data originates from animal model-based studies, and the lack of standardised assessment tools makes it difficult to precisely evaluate analgesic effects in patients.

Clinical studies demonstrate the beneficial effect of JAKi in managing pain in patients with RA. Nevertheless, further research is needed to determine whether JAKi should be used in the early stages of RA treatment, or rather in subsequent lines of treatment. Identifying predictors of treatment response remains an important direction for future research.

## Figures and Tables

**Table 1:** Characteristics and results of the main clinical studies included in the review.

Author, Year	Study Aim	Intervention Groups	Control Groups	Pain Measure	The Impact of Treatment on Pain Control
Taylor P.C. et al., 2022 [40]	To compare the effect and time to achieve a clinically significant pain control for baricitinib (used as monotherapy or in combination with MTX) and MTX used as monotherapy.	1. BARI 4 mg orally once daily (*N* = 159) 2. BARI 4 mg orally once daily + MTX (*N* = 215)	Placebo +MTX from 10 to 20 mg orally once weekly (*N* = 210)	Visual Analogue Scale (VAS); Patient’s Global Assessment of Disease Activity (PtGA)—VAS; Short Form-36 Health Survey (SF-36)—Physical Component Score (PCS);	Baricitinib, used as monotherapy or in combination with MTX, provided a greater and more rapid reduction in pain compared with MTX monotherapy. Treatment with baricitinib was associated with a longer duration of minimal pain or the absence of pain.
Pope J.E. et al., 2022 [41]	To assess the effect of baricitinib on pain reduction in the group of patients with RA, with consideration of opioid use.	1. BARI 4 mg orally once daily + MTX/cDMARD (*N* = 720) 2. BARI 4 mg orally once daily + MTX/cDMARD + opioid users (*N* = 171) 3. BARI 2 mg orally once daily + cDMARD (*N* = 309) 4. BARI 2 mg orally once daily + cDMARD+ opioid users (*N* = 94) 5. Adalimumab 40 mg every 2 weeks + MTX (*N* = 296) 6. Adalimumab 40 mg every 2 weeks + MTX + opioid users (*N* = 34)	1. placebo+ MTX/cDMARD (compared with BARI 4 mg) opioid users (*N* = 153) 2. placebo+ MTX/cDMARD (compared with BARI 4 mg) opioid non-users (*N* = 739) 3. placebo+ cDMARD (compared with BARI 2 mg) opioid users (*N* = 103) 4. placebo+ cDMARD (compared with BARI 2 mg) opioid non-users (*N* = 301) 5. placebo+ MTX (compared with adalimumab 40 mg) opioid users (*N* = 50) 6. placebo + MTX (compared with adalimumab 40 mg) opioid non-users (*N* = 438)	Visual Analog Scale (VAS)	Baricitinib at a dose of 4 mg provided a greater pain reduction compared with placebo, both in patients using opioids and those not using opioid drugs (*p* < 0.05) at all time points. Baricitinib at a dose of 2 mg also showed a greater reduction in pain compared with placebo in the two groups analysed, with the effect observed from week 4 onwards.
Buch M.H. et al., 2025 [42]	To evaluate the efficacy and safety of filgotinib in combination with MTX in patients with a moderately active form of RA and an inadequate response to MTX.	1. FIL 100 mg orally once daily + MTX (*N* = 121) 2. FIL 200 mg orally once daily + MTX (*N* = 104) 3. Adalimumab subcutaneously 40 mg + MTX (*N* = 72)	Placebo + MTX (*N* = 128)	Visual Analog Scale (VAS)	Filgotinib at doses of 100 mg and 200 mg provided a significant reduction in pain severity compared with placebo from week 2 onwards. In weeks 2 and 4 of treatment, in the group receiving filgotinib at 200 mg, a greater reduction in pain severity on the VAS was observed compared with the group receiving adalimumab.
Taylor P.C. et al., 2024 [43]	To compare the effect of filgotinib on pain management, and investigation of the relationship between the therapeutic efficacy of the drug and the response achieved in terms of pain reduction in patients with actively moderate or severe forms of RA.	FINCH 1: 1. FIL 100 mg orally once daily + MTX (*N* = 480) 2. FIL 200 mg orally once daily + MTX (*N* = 475) 3. Adalimumab subcutaneously 40 mg + MTX (*N* = 325) FINCH 2: 1. FIL 100 mg orally once daily + csDMARDs (*N* = 153) 2. FIL 200 mg orally once daily + csDMARDs (*N* = 147) FINCH 3: 1. FIL 100 mg orally once daily + MTX up to 20 mg orally once weekly (*N* = 207) 2. FIL 200 mg orally once daily + MTX up to 20 mg orally once weekly (*N* = 416) 3. FIL 200 mg orally once daily (*N* = 210) 4. MTX up to 20 mg orally once weekly (*N* = 416)	FINCH 1: 1. Placebo + MTX (*N* = 475) FINCH 2: 1. Placebo + csDMARDs (*N* = 148)	Visual Analog Scale (VAS)	Filgotinib reduced pain severity from week 2 of treatment onwards.
Ogdie A. et al., 2020 [44]	To analyze the efficacy of tofacitinib in pain reduction in patients with RA, psoriatic arthritis, or ankylosing spondylitis.	1. Tofacitinib 5 mg orally twice daily + MTX/csDMARDs (*N* = 959) 2. Tofacitinib 10 mg orally twice daily MTX/csDMARDs (*N* = 955)	Placebo + MTX/csDMARDs (*N* = 551)	Patient’s Assessment of Arthritis Pain (PAAP) score (VAS 0–100 mm) Short Form-36 Health Survey (SF-36) Q7: ‘How much bodily pain have you had during the past week?’ Bodily Pain (BP) domain EQ-5D Pain/Discomfort (PD)	Tofacitinib at doses of 5 mg and 10 mg in patients with RA with an inadequate response to csDMARDs (csDMARD-IR) or TNF inhibitors (TNFi-IR) significantly reduced the mean change (LS) on the PAAP scale against the baseline value, compared to placebo. A similar improvement was observed when using the SF-36v2 questionnaire.
Dougados M. et al., 2022 [45]	To evaluate the effect of tofacitinib on residual pain in patients with RA or PsA, who experienced no inflammation.	1. Tofacitinib 5 mg orally twice daily + csDMARD (*N* = 2330) 2. Adalimumab 40 mg subcutaneously every 2 weeks + csDMARD (*N* = 585)	Placebo+ csDMARD (*N* = 673)	Visual Analog Scale (VAS)	Tofacitinib increased the proportion of patients with reduced pain, compared with placebo.
Strand V. et al., 2019 [46]	To assess the effect of upadacitinib on the outcomes reported by patients (PROs) with RA who have shown an inadequate response to csDMARDs.	1. Upadacitinib 15 mg orally once daily + csDMARD (*N* = 221) 2. Upadacitinib 30 mg orally once daily + csDMARD (*N* = 219)	Placebo + csDMARD (*N* = 221)	Patient’s Global Assessment of Disease Activity (PtGA)—VAS Visual Analog Scale (VAS) Short Form 36 Health Survey (SF-36) Bodily Pain (BP) domain Health Assessment Questionnaire-Disability Index (HAQ-DI)	The median time to pain reduction, assessed on the VAS, was 1 week in patients receiving upadacitinib, compared with 4 weeks in the placebo group.
Strand V. et al., 2019 [47]	To compare the effect of upadacitinib on the outcomes reported by patients (PROs) with RA who showed an inadequate response to csDMARDs.	1. Upadacitinib 15 mg orally once daily + csDMARD (*N* = 164) 2. Upadacitinib 30 mg orally once daily + csDMARD (*N* = 165)	Placebo + csDMARD (*N* = 169)	Patient’s Global Assessment of Disease Activity (PtGA)—VAS Visual Analog Scale (VAS) Short Form 36 Health Survey (SF-36) Bodily Pain (BP) domain Health Assessment Questionnaire-Disability Index (HAQ-DI)	UPA 15 mg and 30 mg was more effective in reducing pain than a placebo on the VAS and SF-36 questionnaire.
Strand V. et al., 2021 [48]	To compare the effect of upadacitinib treatment compared with placebo and adalimumab on the patient-reported outcomes (PROs) in the SELECT-COMPARE study in patients with RA with an inadequate response to MTX treatment.	1. Upadacitinib 15 mg orally once daily + MTX (*N* = 651) 2. Adalimumab (ADA) 40 mg subcutaneously once every two weeks + MTX (*N* = 327)	Placebo + MTX (*N* = 651)	Visual Analogue Scale (VAS); Short Form 36 Health Survey (SF-36) Bodily Pain (BP) domain	The time to pain reduction was shorter in patients treated with UPA or ADA, compared with patients receiving a placebo. When using UPA, pain relief was also greater than with ADA.

## Data Availability

No new data were created in this work.
